# Short-Term Refractive Outcomes of Pseudomyopia Induced by Ocular Blunt Trauma

**DOI:** 10.7759/cureus.31751

**Published:** 2022-11-21

**Authors:** Stergios K Chaloulis, Georgios E Mousteris, Konstantinos T Tsaousis

**Affiliations:** 1 Ophthalmology, Volos General Hospital, Volos, GRC

**Keywords:** pseudomyopia, cycloplegia, angle recession, ciliary spasm, blunt ocular trauma

## Abstract

A teenage female patient visited the ophthalmology emergency department reporting blunt ocular trauma from a stretched elastic band, accompanied by blurred vision. At presentation, uncorrected visual acuity (VA) was 6/60 in the affected eye, improving to 6/7.5 with pinhole. A slit lamp examination showed a mild anterior chamber reaction and iridoplegia with pupil shape irregularity. Gonioscopy revealed partial cyclodialysis with angle recession. Fundoscopy revealed focal commotio retinae with blot hemorrhages. B-scan ultrasonography yielded no pathology. Follow-up examination, the day after the injury, included detailed refraction, which showed a myopic shift in the affected eye. Uncorrected VA improved to 6/15 and the patient achieved 6/7.5 with correction. Clinical findings indicated myopia, which resolved within one week from the incident, and refractive error rapidly decreased to prior emmetropic values.

## Introduction

Pseudomyopia occurs when a spasm of the ciliary muscle prevents the eye from focusing in the distance, and in some cases intermittently; this is different from myopia, which is caused by the eye's shape or another basic anatomy. Pseudomyopia may have either organic causes, due to stimulation of the parasympathetic nervous system (common causes include head trauma, encephalitis, intracranial mass, and cerebrovascular disease), or functional causes, as a result of eye strain caused by prolonged near distance focusing (e.g. after continuous hours of studying for a test or overuse of tablets and smartphones). Pseudomyopia may present with one of the following symptoms: blurring of distance vision, asthenopia, headache, eyestrain, photophobia, accommodative esotropia, and diplopia [1,2].

Traumatic myopia is a clinical entity that may occur after ocular blunt trauma [3,4]. It usually affects the injured eye only, or occasionally both eyes, and ranges between -1.00 and -6.00 diopters (D) [3,4]. In most cases, it is sudden onset and transient in character, resolving within a few weeks after the injury, although in some cases, it may follow a longer course until full recovery [5]. The aim of this article is to present the natural course of a pseudomyopia case after blunt ocular trauma.

## Case presentation

An adolescent female patient presented to the emergency ophthalmology department reporting a high-velocity blunt trauma caused by a metal belt clip at the end of a stretched elastic band, accompanied by blurred vision in her right eye. At presentation, uncorrected visual acuity (VA) was 6/60 in the affected eye, improving to 6/7.5 with pinhole (no previous history of refractive error). The young woman had no prior ophthalmological or any other medical history. Slit lamp examination showed no corneal damage, a mild anterior chamber reaction with the presence of inflammatory cells, and partial iridoplegia nasally with pupil shape deformation. Intraocular pressure (IOP) was measured at 10 mmHg. Dilated fundoscopy revealed a clear crystalline lens, normal macula, focal commotio retinae in the nasal, and mid-periphery with blot hemorrhages.

**Figure 1:**
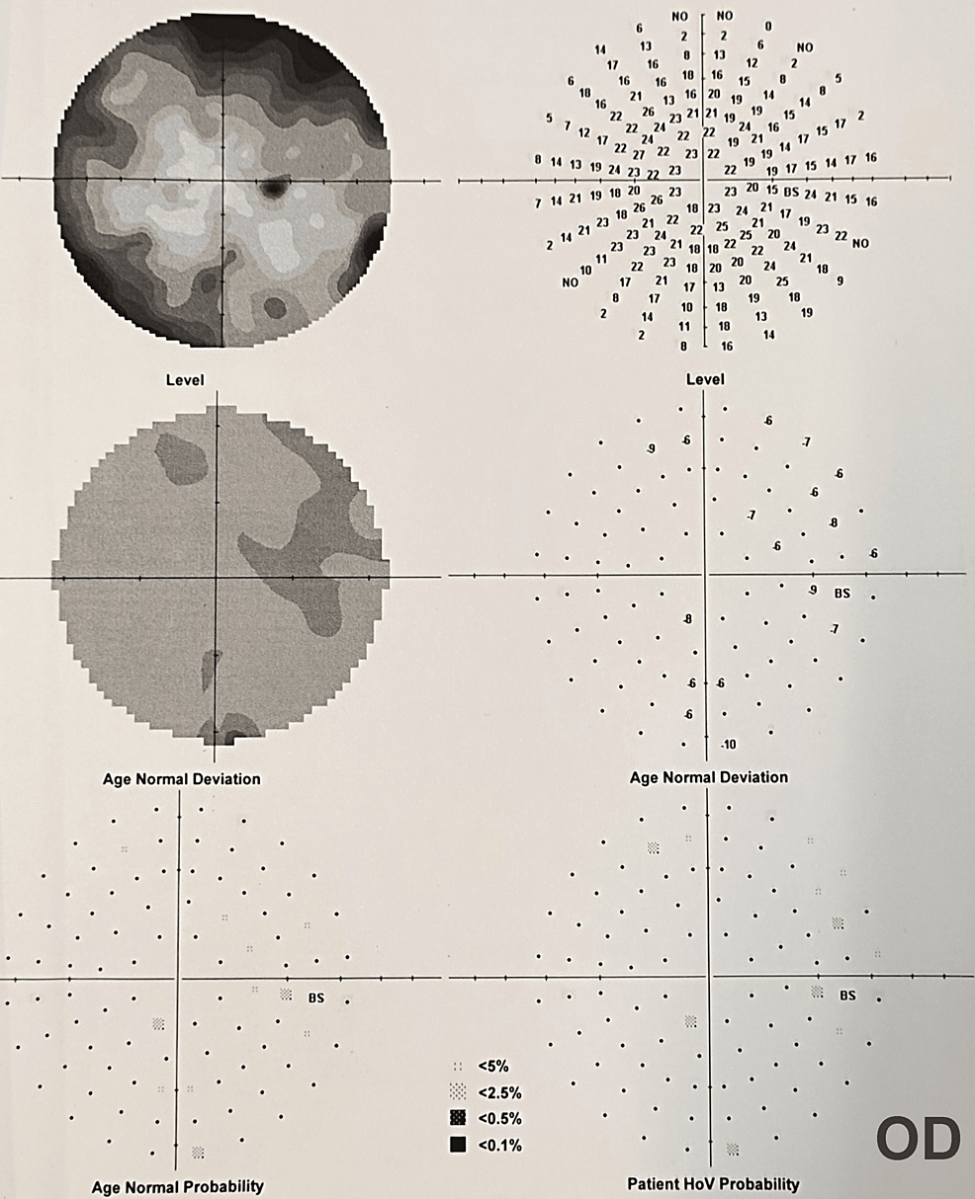
Right eye (OD) visual fields test. An automated perimetry test of the injured eye (OD) showed no pathology.

After the presentation and initial assessment, the patient was referred for B-scan ultrasonography, which showed no pathology. Follow-up examination the day after included detailed auto-refractometry, which showed a myopic shift in the affected eye (right eye (OD): from presumed Plano to -4.50D sphere, -2.00D cylinder x 34°; left eye (OS): -0.50D sphere). Uncorrected VA improved to 6/15 and the patient achieved 6/7.5 with a -5.00D spherical lens correction. IOP was measured at 9 mmHg OD. Gonioscopy revealed cyclodialysis extended in two clock hours range (inferonasally, between three to five hours) with angle recession. We also conducted an automated perimetry visual fields test with normal findings (Figure 1).

Our patient’s chief complaint was the sudden decrease of distant vision following ocular blunt trauma. The absence of any significant pathology along the visual axis ruled out most causes of post-traumatic blurred vision. The onset of myopia in the injured eye despite the patient’s clear ophthalmological history suggested pseudomyopia as the most possible diagnosis.

Topical steroids and cycloplegic drops were prescribed as a treatment. Two days later, when the patient revisited the ophthalmology department, VA in the right eye was further improved to 6/9.5 uncorrected and 6/7.5 corrected. Refractive shift was gradually reduced (OD: -1.25D sphere, -0.50D cylinder x 50°).

**Figure 2:**
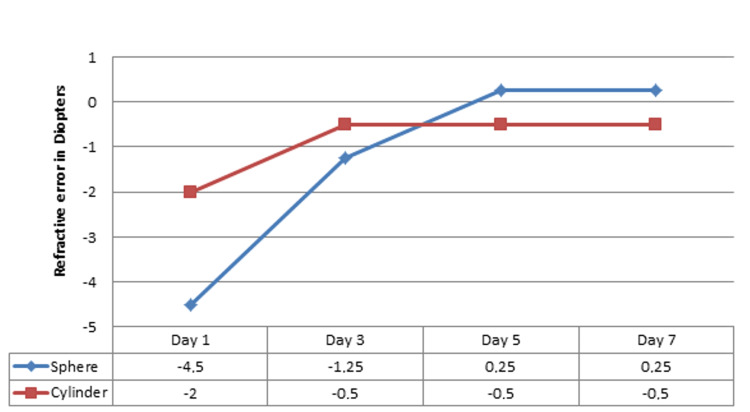
Progression of refractive error in the injured eye (right eye) showing gradual resolution of induced pseudomyopia and astigmatism in the course of a week after the blunt trauma.

**Figure 3:**
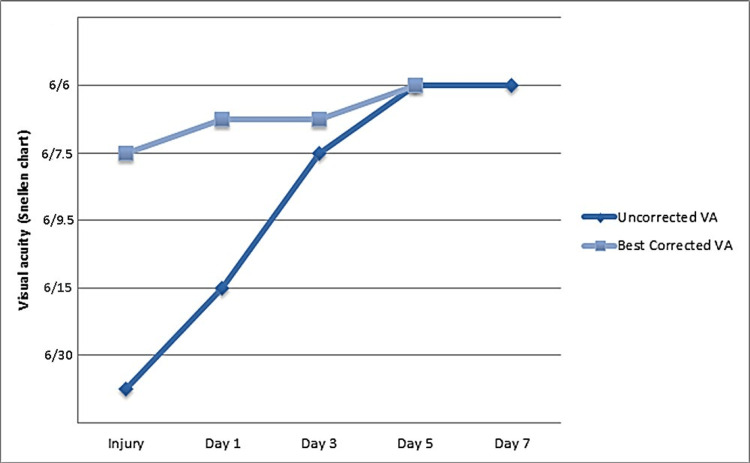
Progression of uncorrected and best-corrected visual acuity (VA) in the patient's left eye following the gradual resolution of pseudomyopia.

The next follow-up, a few days later, showed a complete resolution of pseudomyopia with normal uncorrected VA (OD 6/6 sc). Refraction measurements were as follows: OD: +0.25D sphere, -0.50D cylinder x 53°. IOP had also been raised to 16 mmHg. Figure 2 illustrates the changes in refractive error and Figure 3 shows the improvement of VA over the course of seven days after the trauma.

## Discussion

Many different factors can contribute to the development of pseudomyopia after blunt trauma [3,5,6]. The majority of these factors concern anatomical changes in the ciliary body and the crystalline lens. Ciliary body spasm, ciliochoroidal effusion causing forward displacement of the crystalline lens-iris diaphragm, edema of the ciliary body, and forward shift or axial thickening of the crystalline lens resulting in higher effective power are the most common etiological mechanisms for this condition [5-7]. In some cases, multiple factors result in post-traumatic pseudomyopia. In other cases, only one or a few of these factors are found to cause that effect. Although closed-head traumas may result in decreased visual acuity due to the bilateral spasm of accommodation, one case report describes persistent myopia, still present three months after trauma [8]. In our case, we were not able to determine the exact pathophysiological mechanism liable for the induction of pseudomyopia.

In our patient, the blunt ocular trauma caused a myopic shift of -4.50D, which fully resolved over a span of five to seven days, and a -2.00D astigmatism, probably lenticular, which was reduced after three days to a residual -0.50D and remained stable in the following measurements. It is likely the site of trauma to cause astigmatism at a specific meridian. Pseudomyopia has resolved after five days, possibly due to improvement of topical inflammation. Cycloplegia is suggested to comfort the eye from the ciliary spasm and topical steroids are possibly attributed to inflammation resolution. This probably enhances our hypothesis that in our case pseudomyopia was probably caused by ciliary spasm or edema. Given that further issues like retinal tears or hypotony can occur, patients need frequent follow-ups. In this specific case, the improvement of refraction occurred within less than a week, which according to previous reports is not a general rule [5,6].

Imaging studies such as ultrasound bio-microscopy (UBM) or anterior segment optical coherence tomography (AS-OCT) could assist both the diagnosis and follow-up of injury-induced myopia patients. These modalities reveal important details regarding pathophysiology by depicting anatomical changes of the lens, iris plane, the ciliary body, the anterior chamber depth, and the angle, allowing for precise measurements [3,9]. Anterior segment OCT is generally considered equal to UBM in terms of image quality [9]. It has some advantages over UBM though, as it is easier to perform and does not require contact, causing less discomfort to the patient or possible damage by pressure on the already injured ocular structures. Ultrasound on the other hand could be superior in cases with corneal opacities or hyphema, where the light signal gets blocked [9]. Certainly, they both are not broadly available, thus frequent clinical follow-up is essential for patients with pseudomyopia due to blunt ocular trauma.

## Conclusions

Pseudomyopia is a rare clinical finding in cases of blunt ocular or head trauma. It could involve the injured eye only or both eyes. Post-traumatic pseudomyopia is usually transient, resolving within a few weeks after the injury, although in some cases, it may be long-standing. We presented a case where pseudomyopia followed a significantly shorter course, resolving within a few days. All patients suffering ocular blunt trauma need close monitoring, even when initial findings are not concerning, due to possible late-onset complications, like retinal tears.
